# Evaluation of poly (ADP-ribose) polymerase inhibitor ABT-888 combined with radiotherapy and temozolomide in glioblastoma

**DOI:** 10.1186/1748-717X-8-65

**Published:** 2013-03-19

**Authors:** Lara Barazzuol, Raj Jena, Neil G Burnet, Lisiane B Meira, Jonathan C G Jeynes, Karen J Kirkby, Norman F Kirkby

**Affiliations:** 1 Ion Beam Centre, Faculty of Engineering & Physical Sciences, University of Surrey, Guildford, Surrey, GU2 7XH, UK; 2 Oncology Centre, Addenbrooke’s Hospital, PO Box 193, Cambridge, CB2 0QQ, UK; 3 Department of Oncology, University of Cambridge, Oncology Centre, Addenbrooke’s Hospital, PO Box 193, Cambridge, CB2 0QQ, UK; 4 Department of Biochemistry and Physiology, Faculty of Health & Medical Sciences, University of Surrey, Guildford, Surrey, GU 7XH, UK; 5 Chemical and Process Engineering, Faculty of Engineering & Physical Sciences, University of Surrey, Guildford, Surrey, GU 7XH, UK

**Keywords:** Glioblastoma, PARP inhibition, ABT-888, Radiation, Temozolomide

## Abstract

**Background:**

The cytotoxicity of radiotherapy and chemotherapy can be enhanced by modulating DNA repair. PARP is a family of enzymes required for an efficient base-excision repair of DNA single-strand breaks and inhibition of PARP can prevent the repair of these lesions. The current study investigates the trimodal combination of ABT-888, a potent inhibitor of PARP1-2, ionizing radiation and temozolomide(TMZ)-based chemotherapy in glioblastoma (GBM) cells.

**Methods:**

Four human GBM cell lines were treated for 5 h with 5 μM ABT-888 before being exposed to X-rays concurrently with TMZ at doses of 5 or 10 μM for 2 h. ABT-888^′^s PARP inhibition was measured using immunodetection of poly(ADP-ribose) (pADPr). Cell survival and the different cell death pathways were examined via clonogenic assay and morphological characterization of the cell and cell nucleus.

**Results:**

Combining ABT-888 with radiation yielded enhanced cell killing in all four cell lines, as demonstrated by a sensitizer enhancement ratio at 50% survival (SER_50_) ranging between 1.12 and 1.37. Radio- and chemo-sensitization was further enhanced when ABT-888 was combined with both X-rays and TMZ in the O^6^-methylguanine-DNA-methyltransferase (MGMT)-methylated cell lines with a SER_50_ up to 1.44. This effect was also measured in one of the MGMT-unmethylated cell lines with a SER_50_ value of 1.30. Apoptosis induction by ABT-888, TMZ and X-rays was also considered and the effect of ABT-888 on the number of apoptotic cells was noticeable at later time points. In addition, this work showed that ABT-888 mediated sensitization is replication dependent, thus demonstrating that this effect might be more pronounced in tumour cells in which endogenous replication lesions are present in a larger proportion than in normal cells.

**Conclusions:**

This study suggests that ABT-888 has the clinical potential to enhance the current standard treatment for GBM, in combination with conventional chemo-radiotherapy. Interestingly, our results suggest that the use of PARP inhibitors might be clinically significant in those patients whose tumour is MGMT-unmethylated and currently derive less benefit from TMZ.

## Background

Glioblastoma (GBM), or WHO grade IV glioma, is the most common and malignant of all primary brain tumours, accounting for the most years of human life lost, per patient, than any other form of adult cancer [1]. Despite recent advances in combined modality treatment with surgery, radiotherapy and temozolomide (TMZ) chemotherapy, the outlook for patients is bleak with a median survival of 12–14 months [2].

The key cytotoxic and mutagenic lesion induced by TMZ is considered to be the formation of O^6^-methylguanine (O^6^-MeG). Transcriptional silencing of the repair protein encoded by the O^6^-methylguanine-DNA-methyltransferase (MGMT) gene allows genotoxic damage induced by TMZ to persist, and is predictive of treatment outcome and patient survival [3]. Only 5% of all DNA methylation induced by TMZ occurs at the O^6^ position of guanine. N^7^-methylguanine and N^3^-methyladenine account for 60-70% and 10-20% of the total methyl adducts, respectively. These lesions, together with radiation induced single stranded breaks (SSBs), are recognised and processed by the base excision repair (BER) pathway. The enzyme poly(ADP-ribose) polymerase (PARP) plays a key role in BER, by binding to processed SSBs, and facilitating recruitment of X-ray repair cross-complementing 1 (XRCC1). XRCC1 intervenes as a scaffold protein recruiting other DNA polymerases and DNA ligases.

Recent data suggest that defects in the BER system may have particular impact on the response to both ionizing radiation and TMZ [4]. On this basis, PARP inhibition has been extensively explored as a potential approach to derive additional cytotoxicity from radiotherapy and DNA-methylating agents.

ABT-888 (Veliparib) is a novel, orally bioavailable, and potent PARP inhibitor developed by Abbott laboratories from a modification of a benzimidazole ring. ABT-888 inhibits both PARP-1 and PARP-2 enzymes with an inhibitory constant, Ki, of 5.2 and 2.9 nmol/l, respectively [5]. Preclinical pharmacokinetic studies reported oral bioavailabilty values between 56 to 92% and, more importantly, ABT-888′s ability to cross the blood brain barrier (BBB) with plasma to brain ratio of 3:1 as evaluated in tumour-bearing rats [5].

This study investigates the sensitizing effects of ABT-888 in combination with ionizing radiation and TMZ on four human GBM cell lines. It is the first *in vitro* study to investigate possible synergy between these three agents, and to assess the influence of MGMT promoter methylation status on tumour response.

## Methods

### Cell culture

Four human GBM cell lines (T98G, LN18, U87 and U251) were used in this study. T98G cells were provided by Mick Woodcock, Gray Institute for Radiation Oncology and Biology, Oxford, UK; U87 and U251 cells were obtained from the Health Protection Agency Culture Collections (HPACC, Wiltshire, UK) and LN18 from the American Type Culture Collection (ATCC, Middlesex, UK). All cell lines were confirmed *Mycoplasma* free before use. The cells were cultured as previously described in Barazzuol et al. [6].

### MGMT Western blot analysis

Whole cell lysates were prepared in assay buffer (20 mM Tris, 100 mM NaCl, 5 mM EDTA, 1 mM EGTA, 5 mM β-mercaptoethanol; pH 7.5) and passed repeatedly through a 21-gauge needle for lysis. The cell lysate was then centrifuged at 13,000 g for 20 min at 4°C. Protein concentration was measured using the Bradford assay (Thermo Scientific, Northumberland, UK). Whole-cell lysates (30 μg) were mixed with 5 × SDS loading buffer and boiled for 5 min prior to SDS-PAGE on Bio-Rad precast gel (Bio-Rad, Hertfordshire, UK), run at a constant voltage of 125 V for 1.5 h. Semi-dry transfer was done to PVDF membrane for 30 min, using the Bio-Rad Trans-Blot Turbo transfer system. After blocking in 5% skimmed milk in TBST (20 mM Tris–HCl, 150 Mm NaCl, 0.1% Tween 20; pH 7.6) for 5 h, the membrane was incubated at 4°C overnight with primary antibody against MGMT (2739; Cell Signaling Technology, Danvers, US) at 1:250 dilution in TBST. Bound antibodies were visualised with peroxidase-conjugated goat anti-rabbit IgG (1:2000 in TBST) using the Bio-Rad Immun-Star Western C chemiluminescence kit according to the manufacturer’s instructions.

### Drug treatment

TMZ was provided by Fluka (Sigma-Aldrich, Dorset, UK) and reconstituted in dimethylsulfoxide (DMSO) to a final concentration not exceeding 0.1% (at this concentration, DMSO alone had no effect on cell viability). TMZ was administered at different concentrations and exposure times according to the type of experiment. For single-agent TMZ cytotoxicity, cells were exposed continuously to increasing concentrations of TMZ according to the MGMT status. For combined TMZ, ABT-888 and radiation, TMZ was administered in 5 μM for the MGMT-methylated cells and 10 μM for the MGMT-unmethylated cells for a total exposure time of 2 h, including 1 h before irradiation. After 2 h with TMZ, the medium was replaced.

ABT-888 was supplied by Enzo Life Sciences (Farmingdale, US) and reconstituted in Milli-Q water. For single ABT-888 cytotoxicity, cells were incubated continuously with increasing concentrations of ABT-888 from 0.002 to 50 μM. For the combined experiments with TMZ and radiation, ABT-888 was used at 5 μM and administered for 5 h prior to TMZ treatment and irradiation (2 h exposure time for TMZ).

### Irradiation

X-ray irradiation was performed using a Gulmay machine operating at 250 kVp with a dose rate of 0.65 Gy/min (Royal Surrey County Hospital, Guildford, UK). Cells were grown in 6- well plates and incubated for 5 h before irradiation. Cells were then exposed at room temperature to doses between 1 to 6 Gy.

### Clonogenic survival assay

Clonogenic assay was used to evaluate single drug cytotoxicity (TMZ and ABT-888) and combined treatments (ABT-888, TMZ and X-rays). Cells were grown in 6-well plates and after treatment incubated for up to 14 days. Colonies were fixed with 50% ethanol in PBS and then stained with 5% crystal violet in PBS (Sigma-Aldrich, Dorset, UK). The colonies with more than 50 cells were counted and the survival fractions were determined taking into consideration the plating efficiency for all treatment modalities based on three separate experiments.

### pADPr immunofluorescence quantification

Cells were grown in polystyrene dishes at a concentration of 5 × 10^5^ cells/ml, and pre-treated with 5 μM ABT-888 for 2 h before treatment with 20 mM hydrogen peroxide (H_2_O_2_) for 10 min with or without 5 μM ABT-888. Cells were then washed with ice-cold PBS and fixed with ice-cold methanol/acetone (50:50) for 5 min. Samples were then washed twice with ice-cold PBS and incubated with 1% BSA in PBS for 30 min, before being probed for pADPr adding an anti-pADPr antibody (ab14459; Abcam, Cambridge, UK) at a dilution of 1:400 in 1% BSA in PBS for 1 h at room temperature. Cells were then washed three times with PBS before adding FITC-conjugated goat anti-mouse IgG secondary antibody (Millipore, Watford, UK) at a dilution of 1:400 in 1% BSA in PBS for 1 h protected from light. Cells were washed three times with PBS before adding 2.5 μg/ml 4^′^,6-diamidino-2-phenylindole dilactate (DAPI; Invitrogen, Oregon, US) in PBS for 1 min. Finally, round coverslips were mounted with 10 μl of ProLong Gold anti-fade reagent (Invitrogen, Oregon, US). Samples were analysed using the microscope described in a separate paper [7] at ×40 magnification. Image processing was performed using ImageJ (v1.44p, National Institutes of Health, Bethesda, US) and the pADPr signal intensity was measured as the mean gray value within selected regions of interest (ROI) corresponding to the relative DAPI-stained nuclei.

### Cell death analysis

Cells were treated as described above and collected at different time points after irradiation (1, 5, 10, 24, 48 and 72 h for 3 Gy; and, 24 and 48 h for 2 and 4 Gy) and fixed in 1% formaldehyde (Sigma-Aldrich, Dorset, UK). Samples were stained with 10 μg/ml acridine orange and 8.3 μg/ml Hoechst 33342 (Invitrogen, Oregon, US). The morphological characterization of cell death included apoptosis, necrosis and mitotic catastrophe (Additional file 1). Between 200 and 400 cells were scored for each sample.

### Statistical analysis

All experiments were performed in either duplicate or triplicate. The error bars represent the standard error among the different experiments. The sensitizer enhancement ratio (SER) was used to evaluate the drug-radiation interaction. Its value was estimated from fitting to the Linear-Quadratic (LQ) model as follows:

$$\mathrm{SE}R_{\mathrm{x\%}}=\frac{d_{\mathrm{x\%}}\left(\mathrm{no}\;\mathrm{drug}\right)}{d_{\mathrm{x\%}}\left(\mathrm{drug}\right)}$$

where d_x%_(no drug) is the radiation dose (Gy) required to produce ×% cell survival without drug and d_x%_(drug) in presence of drug (i.e. TMZ and/or ABT-888). SER was calculated at doses related to surviving fractions of 37 and 50%.

Statistical significance was determined using a two-sample t-test and a p value less than 0.05 was considered significant.

## Results

### Cell sensitivity to TMZ and ABT-888

**Figure 1:**
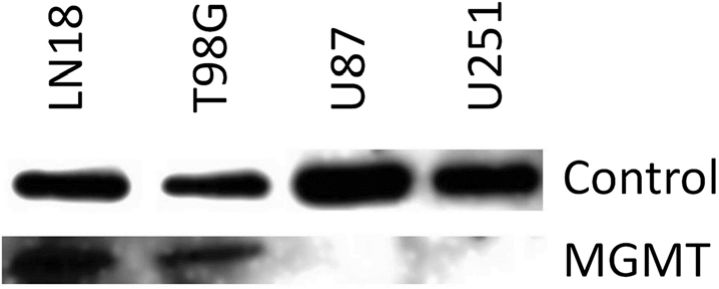
Western blot analysis showing the MGMT protein levels and the protein loading control in a panel of four GBM cell lines (LN18, T98G, U87 and U251).

**Figure 2:**
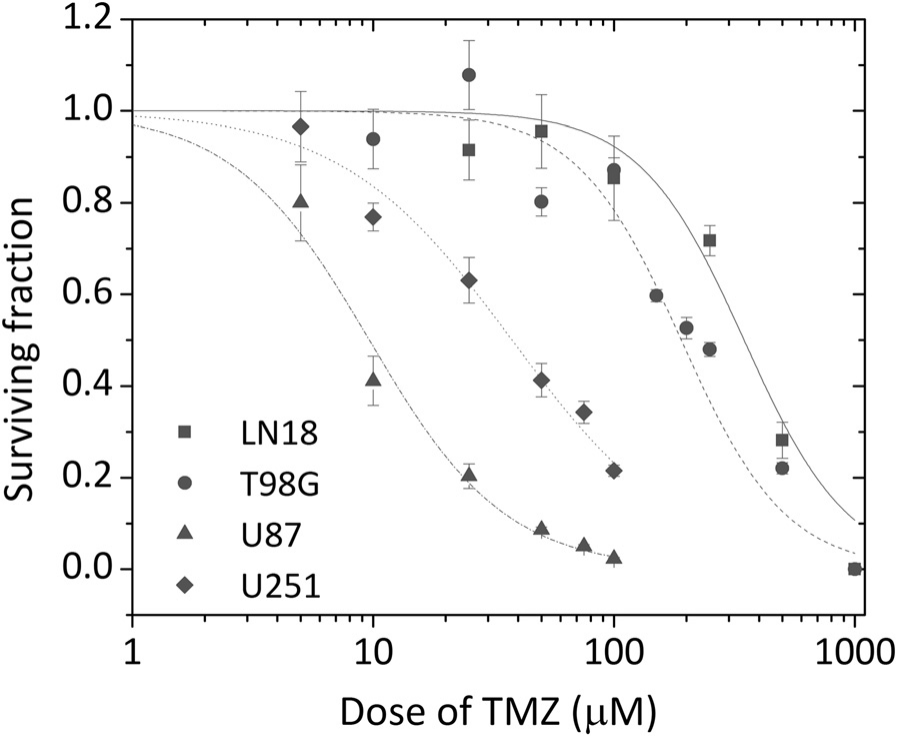
**Cell survival curves of MGMT-unmethylated cell lines, LN18 and T98G, and MGMT-methylated cell lines, U87 and U251.** Cells were exposed continuously to increasing concentrations of TMZ alone. The data were fitted with the Hill equation (solid line) in order to estimate the EC_50_ values. Error bars indicate the standard error of at least three independent experiments.

Our panel of four GBM cell showed heterogeneous MGMT protein expression. LN18 and T98G showed high expression levels of MGMT; whereas, the other two cell lines, U87 and U251, had undetectable levels of MGMT (Figure 1). The EC_50_ values for TMZ ranged between 9.64 (U87) to 346.65 μM (LN18) (Figure 2). MGMT-unmethylated LN18 and T98G cell lines showed the highest resistance to TMZ, confirming that MGMT is an important predictive factor of response to TMZ [3].

**Figure 3:**
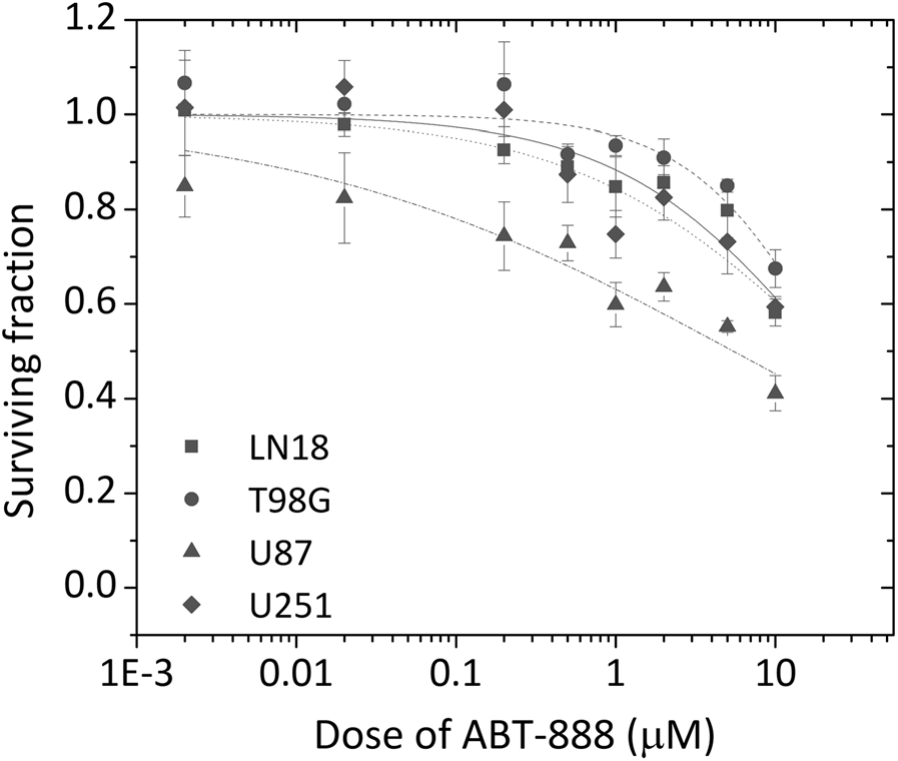
**Cell survival curves of MGMT-unmethylated cell lines, LN18 and T98G, and MGMT-methylated cell lines, U87 and U251.** Cells were exposed continuously to increasing concentrations of ABT-888 alone (0.002-10 μM). The data were fitted with the Hill equation (solid line) in order to estimate the EC_50_ values. Error bars indicate the standard error of at least three independent experiments.

The EC_50_ values for ABT-888 were 19.64, 22.02, 6.44 and 21.9 μM for LN18, T98G, U87 and U251, respectively (Figure 3). No correlation was observed between MGMT methylation status and ABT-888 sensitivity. However, MGMT-methylated, p53 wild type U87 cells were significantly more sensitive to prolonged exposure of ABT-888 than the other cell lines.

**Figure 4:**
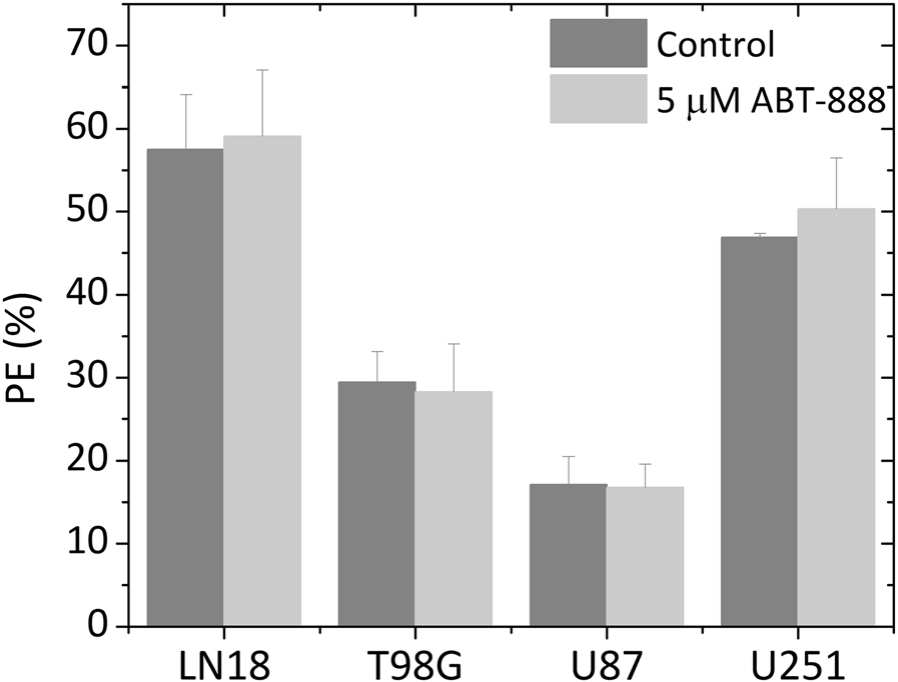
**Plating efficiency (PE) for four GBM cell lines incubated for 5 h with or without 5 μM ABT-888.** No significant difference in survival relative to untreated cells was observed (p > 0.8).

A concentration of 5 μM ABT-888 was then used in the subsequent experiments. This concentration of ABT-888 for an exposure time of 5 h did not yield considerable cellular cytotoxicity (Figure 4).

### Evaluation of pADPr synthesis in the presence of ABT-888

**Figure 5:**
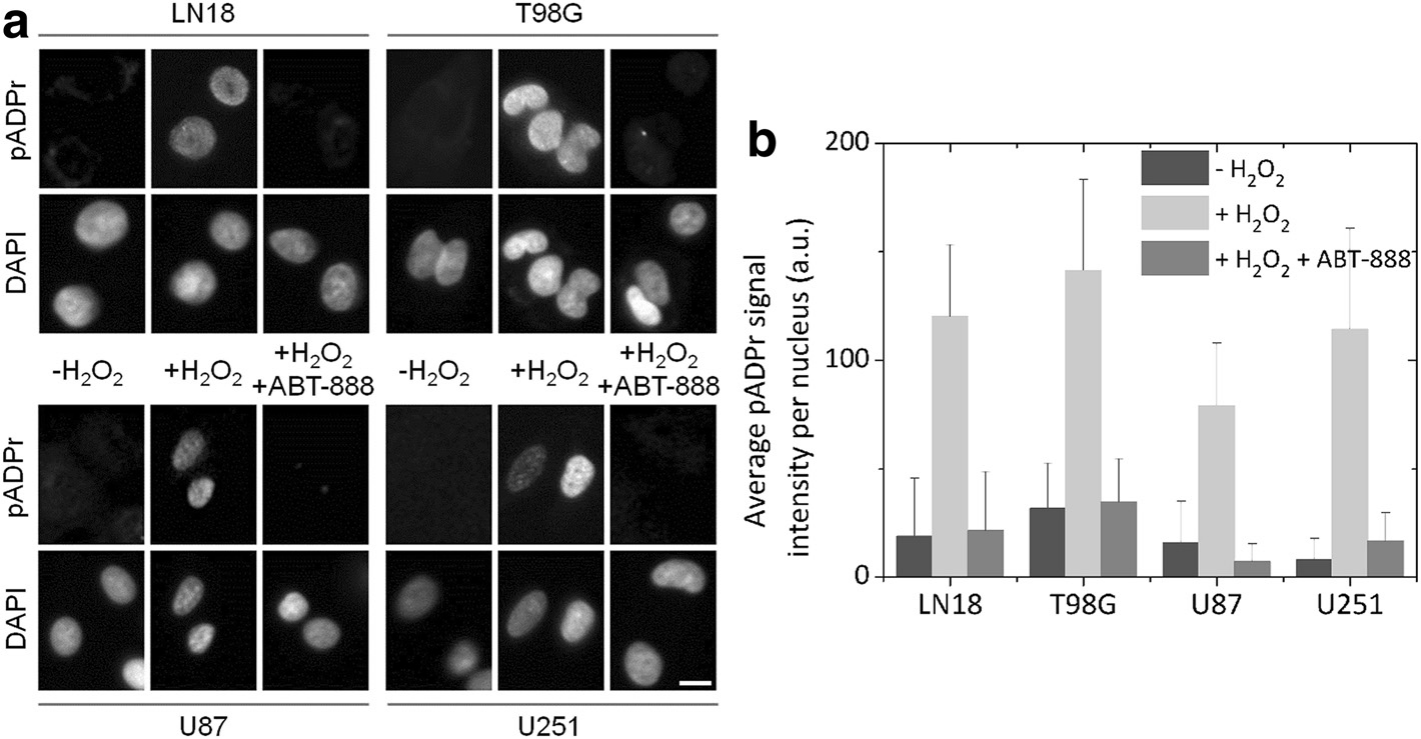
**(a) Immunofluorescence detection of pADPr in GBM cells exposed to 20 mM H**_**2**_**O**_**2 **_**with or without 5 μM ABT-888; scale bar at bottom right = 10 μm.** (**b**) Fluorescence intensity quantification of the pADPr signal estimated using ImageJ (v1.44p, National Institutes of Health, Bethesda, US). Error bars indicate the standard error among 100 cell nuclei.

Upon DNA damage, PARP catalyzes the formation of the polymer pADPr, therefore PARP activity was assessed by measuring the level of pADPr. Under normal conditions, all cells displayed low basal levels of pADPr. Treatment with 20 mM H_2_O_2_ induced PARP activation as demonstrated by a rapid increase of pADPr synthesis. In contrast, cells treated with 5 μM ABT-888 for 2 h before exposure to H_2_O_2_ showed no significant difference in pADPr as compared to basal levels (Figure 5). These data validated that the dose of ABT-888 chosen was suitable to inhibit PARP activity.

### Clonogenic survival after treatment with X-rays, TMZ and ABT-888

**Figure 6:**
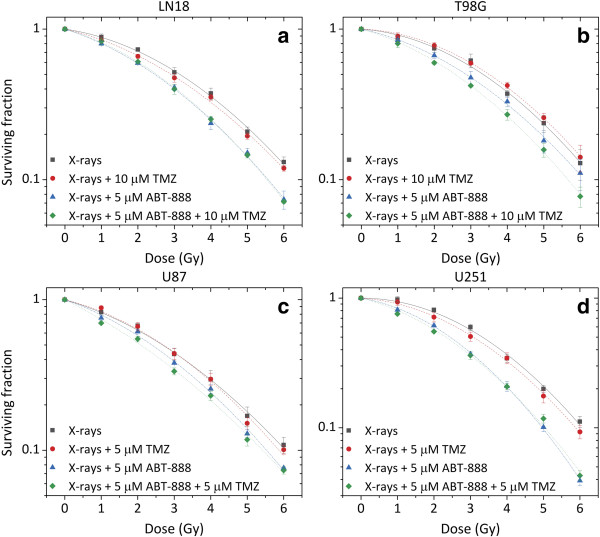
**Cell survival curves of MGMT-unmethylated LN18 (a) and T98G cells (b), and MGMT-methylated U87 (c) and U251 cells (d).** Cells were treated with 5 μM ABT-888 for 5 h before being exposed to either 5 (**c** ,**d**) or 10 (**a**, **b**) μM TMZ for 2 h, including 1 h before and 1 h after irradiation. Symbols represent mean ± standard error of at least three independent experiments.

Cell survival was investigated after combined treatment with ABT-888, TMZ and X-rays (Figure 6). No significant interaction between X-rays and TMZ for the doses chosen (5 and 10 μM for the MGMT-methylated and -unmethylated cell lines, respectively) could be observed in all four cell lines. The SER_50_ and SER_37_ values were near unity and the p values calculated at the dose of 50 and 37% survival were all greater than 0.24 (Table 1).

**Table 1:** Radiobiological parameter values

**Treatment**	**LN18**				**T98G**				**U87**				**U251**			
	α (Gy^-1^)	β (Gy^-2^)	SER_50_ (p)	SER_37_ (p)	α (Gy^-1^)	β (Gy^-2^)	SER_50_ (p)	SER_37_ (p)	α (Gy^-1^)	β (Gy^-2^)	SER_50_ (p)	SER_37_ (p)	α (Gy^-1^)	β (Gy^-2^)	SER_50_ (p)	SER_37_ (p)
**X-rays**	0.08 ± 0.02	0.04 ± 0.01	-	-	0.04 ± 0.02	0.05 ± 0.01	-	-	0.15 ± 0.04	0.04 ± 0.01	-	-	0.01 ± 0.02	0.06 ± 0.01	-	-
**X-rays + 5/10 μM TMZ**	0.13 ± 0.02	0.04 ± 0.01	1.08 (0.32)	1.06 (0.36)	0.02 ± 0.01	0.05 ± 0.01	0.95 (0.52)	0.96 (0.50)	0.12 ± 0.05	0.05 ± 0.01	0.99 (0.93)	1.01(0.92)	0.04 ± 0.03	0.06 ± 0.01	1.08 (0.24)	1.06 (0.30)
**X-rays + 5 μM ABT-888**	0.18 ± 0.02	0.04 ± 0.01	1.28 (0.01)	1.23 (0.01)	0.12 ± 0.02	0.04 ± 0.01	1.16 (0.25)	1.12 (0.20)	0.21 ± 0.02	0.04 ± 0.01	1.13 (0.16)	1.12 (0.17)	0.12 ± 0.02	0.07 ± 0.01	1.37 (0.002)	1.31 (0.002)
**X-rays + 5 μM ABT-888 + 5/10 μM TMZ**	0.16 ± 0.01	0.05 ± 0.01	1.25 (0.02)	1.21 (0.01)	0.15 ± 0.01	0.04 ± 0.01	1.30 (0.03)	1.24 (0.01)	0.33 ± 0.02	0.02 ± 0.01	1.30 (0.03)	1.24 (0.04)	0.20 ± 0.01	0.05 ± 0.01	1.44 (0.003)	1.35 (0.001)

Mean values of α, β, SER_50_ and SER_37_ (including p values) of MGMT-unmethylated LN18 and T98G cells, and of MGMT-methylated U87 and U251 cells, estimated by fitting the cell survival to the LQ model.

The combination of X-rays and ABT-888 led to a substantial radiosensitizing effect with SER_50_ ranging between 1.13 and 1.37, and SER_37_ between 1.12 and 1.31. This was also accompanied by an increase in α parameter of the LQ model (Table 1). The highest radiosensitization was found in LN18 and U251 cell lines, as demonstrated by SER_50_ values above 1.28. T98G and U87 cell lines displayed only a modest effect of ABT-888 on the radiation survival curve (p = 0.16-0.24 Figures 6b and 6c). Importantly, the radiosensitizing effect of ABT-888 was independent of the MGMT methylation status.

The triple combination of X-rays, TMZ and ABT-888 was more effective than single agents in all four cell lines and appeared to be more pronounced in the two MGMT-methylated cell lines. Higher levels of ABT-888-mediated sensitization to X-rays and TMZ were observed in both U87 and U251 cell lines with SER_50_ of 1.30 and 1.44, respectively. Further sensitization was also observed in the MGMT-unmethylated T98G cell line with SER_50_ of 1.30 for all three agents compared to 1.16 with the dual combination of X-rays and ABT-888. However, no additional enhancement was observed with LN18 cells after trimodal treatment compared to X-rays and ABT-888 (SER_50_ of 1.28 compared to SER_50_ of 1.25; Table 1).

### Induction of apoptosis by X-rays, TMZ and ABT-888

**Figure 7:**
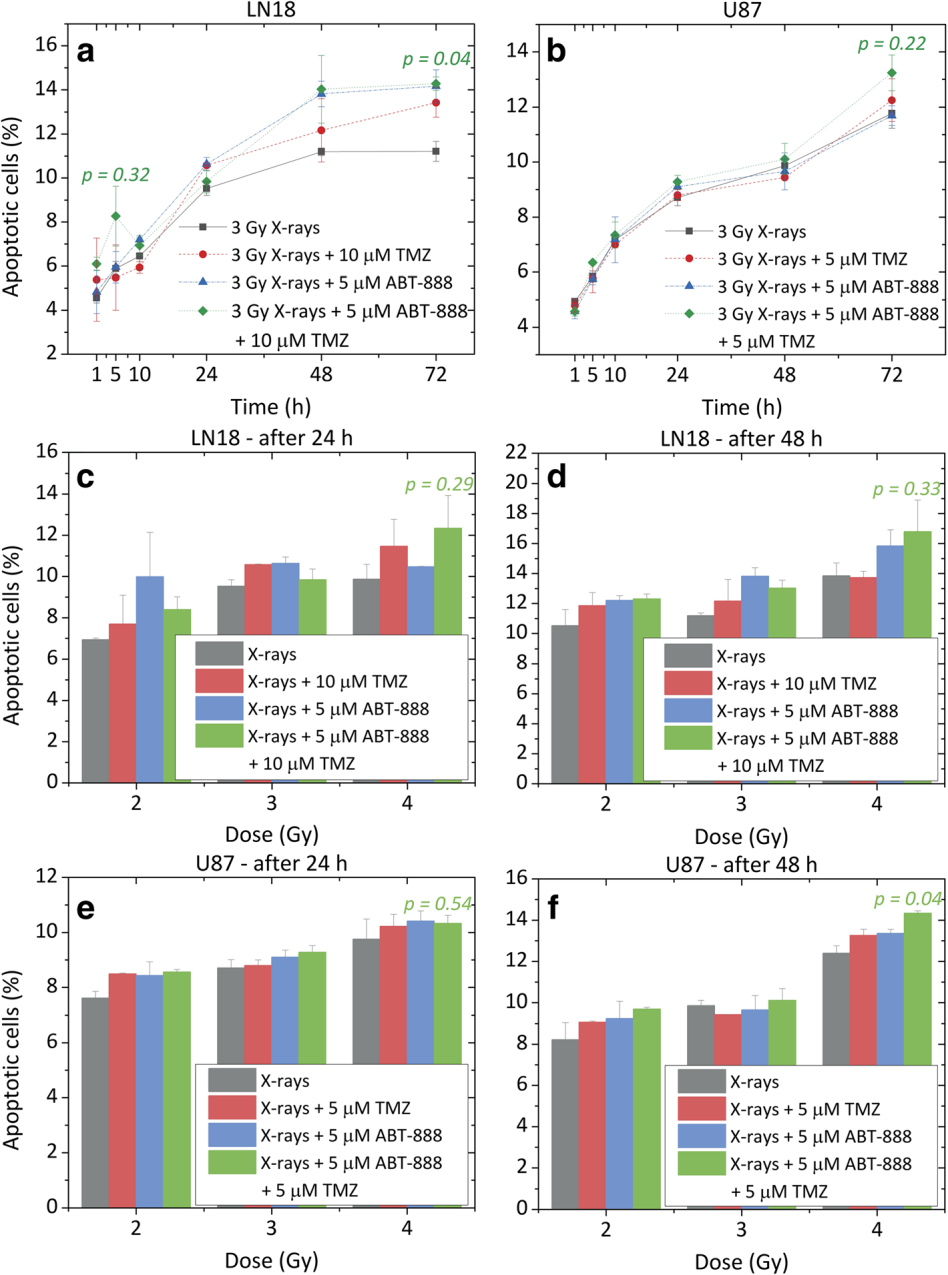
**Percentages of apoptotic cells in MGMT-unmethylated LN18 (a) and MGMT-methylated U87 cells (b) up to 72 h after irradiation.** Cells were exposed for 5 h to 5 μM ABT-888 before being treated with 5 (**b**) or 10 (**a**) μM TMZ for 2 h and irradiated with 3 Gy X-rays. Dose–response percentages of apoptotic cells in MGMT-unmethylated LN18 (**c, d**) and MGMT-methylated U87 cells (**e, f**). Cells were exposed to 2, 3 and 4 Gy X-rays and fixed at 24 and 48 h after irradiation. Error bars indicate the standard error of two independent experiments. Please note the scale change on the Y-axis of the graphs. P values relatively to the control are also shown.

Time-course measurements of apoptosis were undertaken after exposure to a radiation dose of 3 Gy in combination with TMZ and ABT-888 (Figure 7). The induction of apoptosis increased with time reaching a maximum level at 72 h in the range of 11.21 to 14.28% and 11.77 to 13.24% for LN18 and U87 cells, respectively (p<0.04 Figure 7a; p = 0.22 Figure 7b).

In MGMT-unmethylated LN18 cells, a single early apoptotic peak was observed 5 h after treatment with X-rays, TMZ and ABT-888, as demonstrated by the amount of apoptotic cells that was greater (8.27%) than with X-rays alone (5.89%). However, this disproportion was not statistically significant (p = 0.23 Figure 7a). This peak was not seen in the MGMT-methylated U87 cells. Apoptotic responses for U87 cells were very similar for the different treatment combinations (p > 0.05 Figure 7a).

In addition, dose–response measurements were performed at 24 and 48 h after irradiation with 2, 3 and 4 Gy (Figure 7). Both LN18 and U87 cells showed a dose-dependent increase in radiation-induced apoptotic cells. At 48 h, the trimodal treatment seemed to be more effective than single modalities. In particular, in U87 there was a significant difference in apoptosis after treatment with 4 Gy, TMZ and ABT-888 as compared with radiation alone (p < 0.04 Figure 7f).

The sensitizing effect of ABT-888 to TMZ and X-rays may involve other cell death pathways distinct from apoptosis. Therefore, mitotic catastrophe and necrosis were evaluated at 72 h after treatment with 3 Gy X-rays, TMZ and ABT-888. A minimal amount of cells (< 3%) undergoing mitotic catastrophe and necrosis was seen in both LN18 and U87 cells for all treatment combinations (data not shown).

## Discussion

PARP inhibition is a promising mechanism for enhancing efficacy of chemoradiation therapy. A number of PARP inhibitors are currently being assessed in clinical trials, including ABT-888 for which six phase I-II clinical trials exist in patients with brain or central nervous system (CNS) tumours [8].

To date, only one preclinical study has looked at the trimodal combination of PARP inhibitor ABT-888 with TMZ and X-rays in GBM xenografts [9]. The present *in vitro* study suggests that ABT-888 enhances the effects of radiation. A further sensitization has also been shown when ABT-888 was added to both TMZ and X-rays. Although the maximum enhancement in cell killing was obtained in MGMT-methylated cell lines, MGMT expression did not prevent ABT-888-mediated sensitization. This study also indicates that PARP inhibition has an effect on the apoptotic cell death pathway.

### ABT-888 is a potent inhibitor of PARP

Our study confirmed ABT-888′s favourable pharmacokinetic profile and effective attenuation of pADPr formation at a non-cytotoxic concentration of 5 μM in all four GBM cell lines. These results concur with previously reported data by Albert et al. [10] on H460 lung carcinoma cells and Horton et al. [11] on leukaemia cells, in which an optimal dose of 5 μM ABT-888 was determined for *in vitro* models. Importantly, phase 0-I clinical trials have established the achievable area under the plasma concentration time curve for ABT-888 to be 1.46 μM at an initial dose of 10 mg administered orally twice a day (BID) showing that μM concentrations are clinically achievable [12,13].

The EC_50_ values for ABT-888 did not show strong variations among the cell lines, except for the MGMT-methylated, p53 wild-type U87 cell line (EC_50_ = 6.44 μM). It would be of interest to elucidate the relationship between PARP and p53 as all the other cell lines (LN18, T98G and U87) were mutant for p53. Previous reports suggest that PARP-1 is a critical regulator of the p53 response to DNA damage [14,15]. This observation might be relevant to the clinical treatment of GBM as about a third of GBMs have p53 mutations [16].

### ABT-888 enhances radiation response regardless of the MGMT status

The results demonstrated that exposure to 5 μM ABT-888 for 5 h before irradiation resulted in significant radiosen-sitization of all four cell lines (SER_50_ = 1.12-1.37), regardless of the MGMT methylation status (Figure 6). The radiosensitizing effect of ABT-888 seemed to be inversely related to the cell population doubling time. Indeed, this effect was more pronounced in LN18 and U251 cells with SER_50_ of 1.28 and 1.37, respectively, and doubling times of 24 h. This is relevant in the case of brain tumours as the surrounding normal tissue is composed of cells which proliferate slowly or not at all [17-19].

Consistent with the current study, Albert et al. [10] found that 6 h exposure to 5 μM ABT-888 sensitizes lung cancer H460 cells to radiation with a SER_25_ of 1.27. This was also accompanied by a delay in the resolution of γ-H2AX foci at 6 h after irradiation. Similarly, Efimova et al. [20] noted that ABT-888 markedly enhances persistence of γ-H2AX foci in breast cancer cells up to 24 h after irradiation. Liu et al. [21] also showed that ABT-888 impairs the resolution of DSBs remaining at 24 h in the malignant prostate cancer 22RV1 cell line. Altogether, these data suggest that the radiosensitizing effect of ABT-888 is likely to be the consequence of an interaction between unrepaired SSBs and collapsed DNA replication forks [17]. Collapsed replication forks are recognized by the cell cycle checkpoint system which in turn initiates cell cycle arrest, DNA repair or cell death [4].

*In vivo*, one study using an HCT-116 colon model reported that ABT-888 is an effective radio-sensitizer [5]. However, Clarke et al. [9] reported no effect of ABT-888 addition on survival relative to radiotherapy alone on two primary GBM xenografts.

### ABT-*888* further enhances response to TMZ plus X-rays in MGMT-methylated cell lines

Stratification of clinical treatment response by MGMT-methylation status demonstrates poorer outcomes for patients with MGMT-unmethylated tumours. An agent capable of enhancing radiation response in this group would be a valuable new treatment. Our study suggests that trimodal treatment with ABT-888, TMZ and X-rays seems to mostly enhance cell killing in the MGMT-methylated U87 and U251 cell lines (Figure 6). The relative SER_50_ increased from 1.13 and 1.37 with ABT-888 plus X-rays to 1.3 and 1.44 with ABT-888, TMZ and X-rays for U87 and U251 cell lines, respectively. These SER values lie in the range of those obtained with platinum- and taxane-based chemotherapy for different tumour types and end points [22,23].

An increase in SER_50_ was also noted in the MGMT-unmethylated T98G cells. This observation suggests that the MGMT methylation status is not an absolute predictor of response to trimodal treatment. However, there is disagreement in the literature on whether ABT-888-mediated sensitization to TMZ is independent of the MGMT. Palma et al. [24] reported that neither MGMT nor mismatch repair (MMR) precluded sensitivity to ABT-888 plus TMZ in several tumour types. Likewise, Horton et al. [11] suggested that ABT-888 chemo-potentiation in leukaemia and colon cancer cells might not depend on MGMT activity. However, the authors acknowledged that ABT-888 was less effective in the presence of elevated MGMT levels. In contrast, Clarke et al. [9] showed that not all GBM tumours respond equally to ABT-888 plus TMZ, suggesting that ABT-888 may not overcome tumour resistance to TMZ.

Furthermore, it would be of interest to explore different treatment schedules, in particular a different duration of ABT-888 and TMZ exposure before irradiation, and whether TMZ might further sensitize the cells to radiation. To date, a growing number of preclinical studies have looked at the effects of TMZ on the radiosensitivity of GBM cell lines reporting opposing results. While some studies support a synergistic effect between concurrent TMZ and radiation in favour of radiosensitization [25-28], other papers reported independent cell killing [29-32]. It is likely that the optimal schedule of drug administration for TMZ-mediated radiosensitization is the one that will also result in an increased efficiency of ABT-888.

### ABT-888 has an effect on apoptotic response

Apoptosis is an energy dependent form of cell death, and as such, it requires adenosine-5^′^-triphosphate (ATP). The principal substrate of PARP is nicotinamide adenine dinucleotide (NAD+), which is required to catalyse pADPr in the presence of DNA damage. In turn, a reduction in NAD+ leads to a depletion of ATP. By preventing ATP loss, inhibition of PARP should enhance the apoptotic response to genotoxic damage. Our results confirm that the combination of ABT-888 with either radiation or radiation plus TMZ had an effect on the apoptotic response, noticeable at later time points after treatment (Figure 7).

Similarly, Albert et al. [10] assessed apoptosis after treatment with 5 μM ABT-888 and radiation on a lung cancer H460 cell line, reporting a 2.8-fold increase in apoptosis compared to control. Additionally, *in vivo* TUNEL analysis on sections of H460 tumour models showed a 65% increase in apoptosis when ABT-888 was added to radiotherapy. In a separate study, Liu et al. [33] showed that 5 μM ABT-888 co-treatment with the DNA alkylating agent N-methyl-N’-nitro-N-nitrosoguanidine (MNNG) induced activation of caspase-9 and caspase-3 and increased apoptosis in cervical cancer HeLa cells by preventing ATP loss. Nowsheen et al. [34] reported a significant increase in apoptosis when head and neck cancer cells were treated with cetuximab and ABT-888. They hypothesized that apoptosis by PARP inhibition was due to intracellular stress signals, which resulted in the activation of the apoptotic intrinsic pathway. More recently, Huehls et al. [35] reported that ABT-888 promoted apoptosis in ovarian cancer cells treated with 5-fluorodeoxyuridine (FdUrd) but not with 5-fluorouracil (5-FU).

## Conclusions

In summary, this study showed that modulating DNA repair by selectively inhibiting PARP is a potential therapeutic approach to enhance standard treatment in patients with GBM. The most attractive use of PARP inhibitors might be in those patients whose tumour is MGMT-unmethylated and currently derive less benefit from chemo-radiotherapy.

## Competing interests

The authors declare that they have no competing interest.

## Authors’ contributions

LB carried out the experiments, analysis and interpretation of data, and drafted the manuscript. RJ participated in the study conception and design, and drafting of the manuscript. LM participated in the Western Blot analysis and revision of the manuscript. NGB and NFK were involved in the data interpretation and drafting of the manuscript. JCGJ participated in the pADPr immunofluorescence quantification and data interpretation. KJK was involved in the study coordination and drafting of the manuscript. All authors read and approved the final manuscript.

## Supplementary Material

Additional file 1 **Morphological classification of cells after dual fluorescent staining with acridine orange (AO) and Hoechst 33342 (HO).** Representative photographs of apoptosis (**a**), mitotic catastrophe (**b**) and necrosis (**c**). (JPEG 37 kb) Click here for file
