# Supplementary figures and images for: Evaluation of poly (ADP-ribose) polymerase inhibitor ABT-888 combined with radiotherapy and temozolomide in glioblastoma
Source: Radiat Oncol. 2013 Mar 19;8:65. doi: 10.1186/1748-717X-8-65 (PMC3622565; doi:10.1186/1748-717X-8-65)

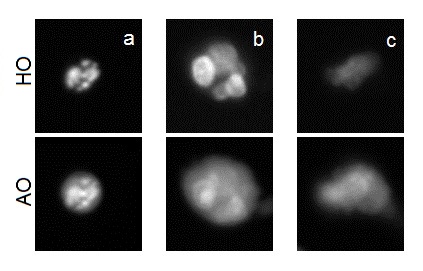

Supplement: Additional file 1 — Morphological classification of cells after dual fluorescent staining with acridine orange (AO) and Hoechst 33342 (HO). Representative photographs of apoptosis (a), mitotic catastrophe (b) and necrosis (c). (JPEG 37 kb) [file 1748-717X-8-65-S1.jpeg]
